# The Influence of Drug–Polymer Solubility on Laser-Induced In Situ Drug Amorphization Using Photothermal Plasmonic Nanoparticles

**DOI:** 10.3390/pharmaceutics13060917

**Published:** 2021-06-21

**Authors:** Nele-Johanna Hempel, Padryk Merkl, Matthias Manne Knopp, Ragna Berthelsen, Alexandra Teleki, Georgios A. Sotiriou, Korbinian Löbmann

**Affiliations:** 1 Department of Pharmacy, Faculty of Health and Medical Sciences, University of Copenhagen, 2100 Copenhagen, Denmark; nele.hempel@sund.ku.dk (N.-J.H.); ragna.berthelsen@sund.ku.dk (R.B.); 2 Department of Microbiology, Tumor and Cell Biology, Karolinska Institutet, 17177 Stockholm, Sweden; padryk.merkl@ki.se (P.M.); georgios.sotiriou@ki.se (G.A.S.); 3 Department of Pharmacy, Bioneer:FARMA, 2100 Copenhagen, Denmark; mmk@bioneer.dk; 4 Department of Pharmacy, Science for Life Laboratory, Uppsala University, 75123 Uppsala, Sweden; alexandra.teleki@scilifelab.uu.se

**Keywords:** oral drug delivery, in situ drug amorphization, polymers, amorphous solid dispersion, laser radiation, plasmonic nanoparticles, pharmaceutical nanotechnology

## Abstract

In this study, laser-induced in situ amorphization (i.e., amorphization inside the final dosage form) of the model drug celecoxib (CCX) with six different polymers was investigated. The drug–polymer combinations were studied with regard to the influence of (i) the physicochemical properties of the polymer, e.g., the glass transition temperature (*T*_g_) and (ii) the drug–polymer solubility on the rate and degree of in situ drug amorphization. Compacts were prepared containing 30 wt% CCX, 69.25 wt% polymer, 0.5 wt% lubricant, and 0.25 wt% plasmonic nanoparticles (PNs) and exposed to near-infrared laser radiation. Upon exposure to laser radiation, the PNs generated heat, which allowed drug dissolution into the polymer at temperatures above its *T*_g_, yielding an amorphous solid dispersion. It was found that in situ drug amorphization was possible for drug–polymer combinations, where the temperature reached during exposure to laser radiation was above the onset temperature for a dissolution process of the drug into the polymer, i.e., *T*_DStart_. The findings of this study showed that the concept of laser-induced in situ drug amorphization is applicable to a range of polymers if the drug is soluble in the polymer and temperatures during the process are above *T*_DStart_.

## 1. Introduction

In situ drug amorphization is a drug delivery approach, where a crystalline drug is converted into its amorphous form, e.g., in the form of an amorphous solid dispersion, in the final dosage form. This conversion, i.e., the in situ drug amorphization, may take place immediately after the manufacturing of the final dosage form or directly before administration. Utilizing in situ drug amorphization, downstream manufacturing challenges of amorphous powder, e.g., poor flowability and/or stability issues during storage, such as amorphous–amorphous phase separation, can be circumvented [1,2,3,4,5,6,7].

Successful in situ drug amorphization has previously been described by various methods, such as water immersion [8] and the use of microwave radiation [1,2,3,4,5,9] and laser radiation [10]. The latter two methods utilize electromagnetic radiation sources and were reported to lead to complete amorphization of a compact containing 30 wt% celecoxib (CCX) and the polymer polyvinylpyrrolidone (PVP12) within relatively short time periods, i.e., 10 min of exposure to microwave radiation [2] and 3 min of exposure to laser radiation [10].

It has been suggested that microwave-induced in situ drug amorphization follows a dissolution process of the drug into the polymer at temperatures above the glass transition temperature (*T_g_*) of the polymer. Thus, in accordance with the Noyes–Whitney equation, describing the dissolution rate of a solute into a solvent [11], a smaller drug particle size [2], a higher temperature reached during exposure to microwave radiation, and a lower viscosity of the polymer [12] have been demonstrated to be advantageous for in situ drug amorphization. Microwave-induced in situ drug amorphization is dependent on the presence of an enabling (dielectric) excipient inside the compact that absorbs the microwave radiation and consequently causes a temperature increase inside the compact [13]. So far, sorbed water, inorganic crystal hydrates, glycerol, and polyethylene glycol have been used as enabling excipients [2,3,9,12]. However, previous studies have shown that large amounts of these dielectric excipients are necessary inside the compact to enable complete microwave-induced in situ drug amorphization [2,3,9,12]. For example, approx. 20 wt% sorbed water was necessary to obtain complete amorphization of CCX in PVP12 [2]. In fact, the enabling excipient also functions as a plasticizer of the polymer, i.e., it lowers the polymer *T*_g_ to temperatures that can be achieved upon exposure to microwave radiation (~100 °C). In connection with the *T*_g_, a relatively low molecular weight (*M*_w_) of the polymer has also been shown to be necessary to achieve a high degree of in situ drug amorphization, e.g., the use of PVP12 (*M*_w_ = 2500 g/mol) yielded a higher degree of amorphization compared to PVP17 (*M*_w_ = 9000 g/mol) [5]. The limitations of the temperature reached upon exposure to microwave radiation in relation to the *T*_g_ and *M*_w_ of the polymer, combined with the need for a high amount of dielectric excipient, have so far led to only four reported cases of complete in situ drug amorphization upon exposure to microwave radiation, namely CCX in PVP12 using sorbed water or sodium dihydrogen phosphate mono- or dihydrate as an enabling excipient, CCX in polyethylene glycol 3000 and 4000 using polyethylene glycol as the enabling excipient, and indomethacin in Soluplus^®^ using glycerol as the enabling excipient [2,3,9,12].

With the concept of laser-induced in situ drug amorphization, it is possible to reduce the amount of enabling excipient needed inside the compact, as well as the total exposure time. Furthermore, higher temperatures (up to 150 °C) upon exposure have been reached compared to the use of microwave radiation [10], which can potentially enable the amorphization of more drug–polymer combinations. Using laser radiation, heating of the compacts is achieved by introducing silver plasmonic nanoparticles (PNs), which absorb laser radiation in the near-infrared (near-IR) spectrum. PNs exhibit photothermal properties, i.e., they convert light into heat [14]. The optical extinction of silver PNs was tuned to extend into the near-IR spectrum by adapting the interparticle distance of PNs using a dielectric spacer (SiO_2_) [14,15].

In this study, the silver PNs were obtained by flame spray pyrolysis (FSP) [16,17,18]. Using PNs at 0.1 wt% or 0.25 wt%, laser-induced in situ drug amorphization was successfully obtained for CCX in combination with PVP12 [10]. In the before-mentioned study, it was shown that increasing laser intensity as well as increasing PN load led to a faster temperature increase and a higher maximum temperature, resulting in a faster rate and higher degree of amorphization [10]. This proof-of-concept study was, however, limited to a single polymer, namely PVP12, which has also been successfully amorphized using microwave radiation [2,9,10]. Compacts exposed to laser radiation became fully amorphous after only 3 min compared to exposure to microwave radiation, for which 10 min of exposure was needed to achieve complete amorphization.

It is still unclear whether the concept of in situ drug amorphization is applicable to different types of polymers, e.g., polymers with different drug solubilities as well as different *M*_w_ and *T*_g_. Polymers with a high *T*_g_ cannot be used for microwave-induced in situ drug amorphization, as the temperatures reached during exposure to microwave radiation are not (or not sufficiently) above the *T*_g_ of the polymer. This is because the polymer is only mobile enough to allow for drug dissolution, within a reasonable timeframe, at temperatures above the *T*_g_ of the polymer [5]. Here, the use of PNs can be beneficial to achieve sufficient heating: by using laser-induced in situ drug amorphization, higher temperatures can be reached [10]. It is important to show the applicability of laser-induced in situ drug amorphization for a range of polymers, as this would allow for widening the general approach of radiation-induced in situ amorphization as well as using specific polymers that are suitable for the drug candidate rather than choosing a suitable polymer for the in situ amorphization.

In this study, it was investigated whether the concept of laser-induced in situ drug amorphization is applicable to six different types of polymers commonly used as pharmaceutical excipients, namely; Soluplus^®^ (Soluplus), Kollidon^®^ VA64 (VA64), Shin-Etsu AQOAT^®^ (HPMCAS), Eudragit^®^ EPO (EPO), Eudragit^®^ EL 100 (EL100), and Parteck^®^ MXP (PVA). These polymers cover a range of properties, e.g., they have different *T*_g_, *M*_w_, and solubilities of the drug CCX. CCX was chosen as a model drug, as it was previously successfully used for microwave- and laser-induced in situ amorphization with the polymer PVP. This allowed studying the effect of the polymer type and the polymer properties on the laser-induced in situ drug amorphization, as well as the influence of the drug solubility in the polymer on the successful amorphization.

## 2. Materials and Methods

### 2.1. Materials

Celecoxib (CCX, *M*_w_ = 381.4 g/mol) and magnesium stearate (MgSt, *M*_w_ = 591.3 g/mol) were purchased from Fagron Nordic A/S (Copenhagen, Denmark). Kollidon^®^ VA64 (VA64, polyvinylpyrrolidone-vinyl acetate copolymer, *M*_w_ = 38,200 g/mol) and Soluplus^®^ (Soluplus, polyvinyl caprolactam-polyvinyl acetate-polyethylene glycol graft copolymer, *M*_w_ = 118,000 g/mol) were kindly supplied by BASF (Ludwigshafen, Germany). Shin-Etsu AQOAT^®^ (HPMCAS, hypromellose acetate succinate, *M*_w_ = 18,000 g/mol) was received as a gift from Shin-Etsu Chemical Co., Ltd. (Tokyo, Japan). Eudragit^®^ EPO (EPO, Amino methacrylate copolymer, *M*_w_ = 47,000 g/mol) and Eudragit^®^ EL 100 (EL100, anionic methacrylic acid methyl methacrylate copolymer, *M*_w_ = 125,000 g/mol) were supplied as a gift from Evonik Nutrition & Care GmbH (Darmstadt, Germany). Parteck^®^ MXP Polyvinyl alcohol (PVA, *M*_w_ = 26,300 g/mol) was a gift from Merck KGaA (Darmstadt, Germany).

Silver acetate (99.8% anhydrous) was purchased from Alfa Aesar (Kandel, Germany). Hexamethyldisiloxane (≥98%), acetonitrile (99.8% anhydrous), and 2-ethylhexanoic acid (99%) were purchased from Sigma-Aldrich (Stockholm, Sweden). The oxygen gas for the flame spray pyrolysis synthesis (FSP) was from Strandmøllen (Ljungby, Sweden).

Ethanol (>99.7%, HPLC grade) was purchased from VWR International (Leuven, Belgium). Purified water used for the mobile phase in the HPLC experiments was prepared using a MilliQ water system from LabWater (Los Angeles, CA, USA). Silica gel with indicator (orange gel) as a granulate was purchased from Merck KGaA (Darmstadt, Germany). All chemicals were used as received.

### 2.2. Plasmonic Nanoparticle Synthesis

The silver–silicon dioxide PNs were synthesized by FSP [19] as introduced by Sotiriou et al. 2011 [18] with a target composition of 98 wt% Ag and 2 wt% SiO_2_. The detailed procedure can be found in Hempel et al. (2021) [10]. In short, the dissolved precursors were dispersed at a rate of 5 mL/min into a fine spray by oxygen gas flowing at 5 L/min. This spray was ignited by a methane/oxygen annular support flamelet. The PNs were then collected on a filter above the flame.

### 2.3. Compact Preparation

Firstly, physical drug–polymer mixtures were prepared by mortar and pestle containing 30 wt% CCX, 69.25 wt% polymer, 0.25 wt% PNs, and 0.5 wt% magnesium stearate (lubricant). Using 50 ± 2 mg of the physical mixture, flat-faced compacts with a diameter of 6 mm were obtained by using an instrumented single punch tablet press GTP-1 from Gamlen Instruments (Nottingham, UK). The compaction pressure was set to 160 MPa using a 500 kg load cell (CT-500-022). The compacts were stored over dried silica until further use.

### 2.4. Exposure to Laser Radiation

Laser-induced in situ amorphization was conducted using laser radiation at a wavelength of 808 nm. Table 1 shows an overview of the exposure times used for the different compact compositions. On the laser outlet, a tophat diffuser with a squared profile from Thorlabs Inc. (Mölndal, Sweden) was mounted to evenly distribute the radiation over the compact. The laser output power was adjusted and controlled using a laser diode controller Model ADR 1860 from Shanghai Laser & Optics Century Co., Ltd. (Shanghai, China). Each compact was located on a cover glass slide and elevated from the bottom. The laser intensity used was 1.71 W/cm^2^ distributed over an area of 1.54 cm^2^ as measured at the glass coverslip. Additionally, a cover glass slide was placed on top of the compact to control the formation of a water gas bubble due to evaporation. The cover glass slide had no influence on the in situ drug amorphization (data not shown) (see also Hempel et al. (2021) for more information [10]).

Using an IR thermal camera Testo 871 from Testo SE & CO. KGaA (Lenzkirch, Germany), surface temperature measurements of the compacts were performed during exposure to laser radiation. The IR thermal camera created thermal images, which were saved by the thermography app (version 2.7.0.1803, Testo SE & Co. KGaA, Lenzkirch, Germany) and analyzed using the Testo IRSoft Software (version 4.5, Testo SE & Co. KGaA, Lenzkirch, Germany). Approximately every 6th second, a thermal image was taken of the compact during exposure to laser radiation. Each experiment was conducted in triplicate (*n* = 3).

### 2.5. Water Content Determination

The water content was determined for pure compounds, physical mixtures for the compacts, and the powdered compacts (using mortar and pestle), before and after exposure to laser radiation. For this, a Discovery thermogravimetric analyzer 1 (TGA) from TA Instruments Inc. (New Castle, DE, USA) was used. The TGA experiments were performed under a nitrogen gas atmosphere for which the gas flow was set to 25 mL/min. The weight loss equivalent to the water content was determined using the TA Instruments TRIOS software (version 5.1.1, TA Instruments Inc., New Castle, DE, USA).

Using a heating rate from ambient temperature to 150 °C of 10 °C/min, the water content was determined. All experiments were performed as a duplicate (*n* = 2) apart from compacts exposed to laser radiation. For compacts exposed to laser radiation, for each exposure time and compact composition, the water content was determined in a single run (*n* = 1).

### 2.6. Thermal Analysis

Thermal analysis of samples was performed by differential scanning calorimetry (DSC) using a Discovery DSC from TA Instruments (New Castle, DE, USA). The experiments were performed under a nitrogen gas atmosphere achieved by a gas flow of 50 mL/min into the DSC cell. The data were analyzed using the TRIOS software (version 5.1.1, New Castle, DE, USA) from TA Instruments.

#### 2.6.1. Determination of the Onset Temperature for the Dissolution Process

Using a mortar and pestle, 100 mg physical mixtures containing 30 wt% CCX in each polymer were prepared. Of each physical mixture, 2–4 mg was weighed into a Tzero aluminum pan and sealed with a perforated hermetic lid. The onset of dissolution was determined in the total heat flow using a modulated DSC (mDSC) run with a heating rate of 3 °C/min from 20 to 190 °C. The modulation had an amplitude of 1 °C/50 s (*n* = 2). The sample mass was corrected for the water content of the polymer (see Section 2.5.).

#### 2.6.2. Determination of the Drug–Polymer Solubility

The solubility of CCX was determined in each polymer except for Soluplus, as raw data in that case was available in the literature from Knopp et al. (2016) [20]. For the solubility measurements, 100 mg physical drug–polymer mixtures were made for each drug–polymer combination with 70–90 wt% CCX in 5 wt% increments. Subsequently, 3–5 mg of each mixture and pure CCX were weighed into Tzero aluminum pans, which were sealed with a perforated hermetic lid. The samples were equilibrated at 20 °C for 2 min. Afterwards, a temperature ramp of 1 °C/min to 180 °C was applied (*n* = 2). Using the Flory–Huggins approach, the solubility of the drug in the polymer was calculated from the onset of the dissolution endotherm. The method is described in more detail in Knopp et al. (2015) [21]. The sample mass was corrected for the water content of the polymer (see Section 2.5.).

#### 2.6.3. Glass Transition Temperature of the Polymers

The *T*_g_ of the polymers was also determined by DSC. For each polymer, two *T*_g_s were determined: the *T*_g_ of the bulk polymer (*T*_g_1) and the water-free *T*_g_ (*T*_g_2). Of each polymer, 3–5 mg was weighed into Tzero aluminum pans with a hermetically sealing lid. A modulated DSC (mDSC) run was applied with an amplitude of 1 °C/50 s at a heating rate of 3 °C/min. For the determination of the *T*_g_2, the lid was perforated, and the sample was first heated to 120 °C to allow the water to evaporate, followed by an isothermal period of 10 min before equilibrating to 20 °C. Depending on the polymer, the sample was heated to 140–180 °C for the determination of *T*_g_2. For the determination of *T*_g_1, the sample was not heated higher than 120 °C as described above (no perforation of the lid). All *T*_g_s were determined as the midpoint of the step change. Each experiment was conducted in duplicate (*n* = 2).

### 2.7. Solid-State Characteristics

Solid-state characteristics were determined by diffractometry. For this, X-ray powder diffraction (XRPD) was performed and used to determine the solid-state characteristics of the pure substances (data not shown), physical mixtures for the different compact compositions (data not shown), and compacts before and after exposure to laser radiation. XRPD was performed on a Rigaku MiniFlex from Rigaku Americas Holding Company Inc. (Austin, TX, USA), which was equipped with a Cu Kα radiation source. Approximately 5–10 mg of sample was used, which was then placed on a low background sample holder and scanned from 5–30° 2theta at a speed of 5°/min and no spin. The XRPD was set to a power output of 40 kV and 15 mA. The obtained diffractograms were visually analyzed using the MiniFlex guidance software (version 3.0.2.4, Rigaku Americas Holding Company Inc., Austin, TX, USA), and the raw data were exported to Origin for further analysis.

### 2.8. Quantification of Drug and Qualification of Degradation Using Liquid Chromatography

High-performance liquid chromatography (HPLC) was conducted to quantify the amount of CCX in the compacts before and after exposure to laser radiation. As a representative compact, only the compacts at the respective longest exposure time to laser radiation were measured by liquid chromatography. The HPLC experiments were conducted with a 1260 Infinity HPCL from Agilent Technologies, Inc (Santa Clara, CA, USA) using a reverse-phase Luna 5U C18(2) 100 A column (150 mm × 4.6 µm) from Phenomenex Ltd. (Aschaffenburg, Germany). The chromatography was performed at ambient temperature. The mobile phases were degassed before use.

The HPLC method used in this study was previously reported for the quantification of CCX by Hempel et al. [10]. The original published method was from Dhabu et al. [22] and modified by Hempel et al. [10]. The mobile phases were purified water and ethanol, which were eluted at a ratio of 3:7 (*v*/*v*) at a flow rate of 1 mL/min. From the HPLC vial containing the dissolved drug CCX, a sample volume of 10 µL was injected into the column. The UV detection of CCX was performed at an absorbance maximum at a wavelength of 251 nm. None of the polymers showed absorbance at the chosen wavelength, which was determined by UV spectroscopy prior to the HPLC experiments (data not shown). The retention time of CCX was experimentally found at 2.6 min. According to the literature by Dhabu et al., degradation products would elute at lower retention times than CCX [22].

The samples were prepared by dispersing an amount of the powdered compacts (before or after exposure to laser radiation) in the organic mobile phase ethanol to dissolve and extract CCX. After shaking, the dispersion was filtered using a nylon syringe filter Q-max^®^ RR 25 mm with a pore size of 0.45 µm from Frisenette Aps (Knebel, Denmark), and the first 1 mL was discharged. The sample mass was corrected by the water content of the compact or mixture (see Section 2.5.). The standard curve used to quantify the amount of CCX in the experiments is published in [10] and is usable at a concentration range from 2 to 12 µg/mL, i.e., the samples were diluted accordingly to lie in the concentration range of the standard curve.

## 3. Results and Discussion

Laser-induced in situ drug amorphization has previously been shown feasible for the drug–polymer combination of CCX and PVP12 [10]. Using the same drug, laser-induced in situ amorphization in the current work was attempted using six different polymers with different *T*_g_ and *M*_w_, as well as different drug–polymer solubilities. By discussing the results in light of the rate and degree of amorphization, with respect to the temperature measured, as well as the drug–polymer solubility, conclusions regarding the suitability of certain types of polymers for laser-induced in situ drug amorphization were drawn.

### 3.1. Drug–Polymer Solubility

**Figure 1:**
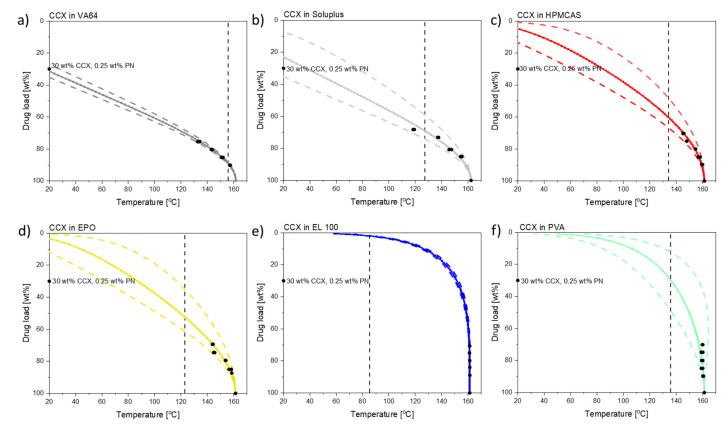
Drug–polymer solubility curves: (**a**) CCX in VA64; (**b**) CCX in Soluplus [20]; (**c**) CCX in HPMCAS; (**d**) CCX in EPO; (**e**) CCX in EL100; and (**f**) CCX in PVA. The black dots indicate the data points used for the calculations and the chosen drug load of 30 wt% used in this study. The vertical black dashed lines indicate the average maximum temperature (*T*_max_) achieved during the longest exposure to laser radiation.

As described in the introduction, laser-induced in situ drug amorphization is a temperature-dependent process. The temperature reached during exposure to laser radiation limits the amount of drug that can dissolve into the mobile polymer. As the in situ drug amorphization will also be limited by the solubility of the drug in the polymer, it is important to determine the solubility of CCX in the six different polymers. To determine the solubility of CCX in the tested polymers, the “dissolution” method was used [23,24]. Figure 1 shows the solubility of CCX in the respective polymers from 20 °C to the melting point of CCX. Table S1 summarizes the predicted values including confidence intervals for the solubility of CCX in the respective polymers at 20 °C (room temperature).

As can be seen in Figure 1, CCX has the highest solubility at room temperature in VA64 and Soluplus (31.8 wt% and 22.5 wt%, respectively). The drug load in the compacts (30 wt%) was below the solubility in VA64 at room temperature and above the solubility in Soluplus at room temperature.

CCX has a low solubility at room temperature in HMCAS and EPO, with 5.3 wt% and 3.6 wt%, respectively, and negligible solubility in EL100 and PVA. As the solubility of CCX in EPO and HPMCAS increases with increasing temperature, it should, in theory, be possible to dissolve 30 wt% CCX in the polymers upon exposure to laser radiation depending on the temperatures reached during exposure.

### 3.2. Laser-Induced In Situ Drug Amorphization

**Figure 2:**
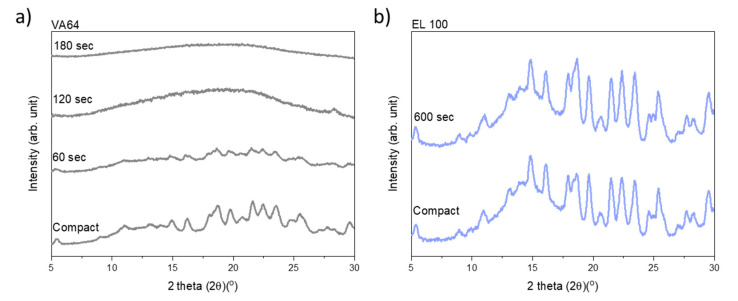
Diffractograms of the compacts before and after different exposure times to laser radiation: (**a**) VA64 compacts (dark grey); (**b**) EL100 compacts (blue).

Immediately after exposure to laser radiation, the compacts were analyzed by XRPD to follow the amorphization process qualitatively. Figure 2 shows the diffractograms for the compacts containing VA64 and EL100 (data for the remaining drug–polymer combinations are available in the Figure S1). As can be seen, upon increasing exposure to laser radiation, the crystalline peaks gradually disappear for the compacts containing VA64 until a fully amorphous halo was obtained after 180 s (Figure 2a). In contrast, the peak intensity of CCX did not decrease for compacts containing EL100, indicating little to no amorphization upon exposure to laser radiation for 600 s (Figure 2b). The exposure times to reach complete amorphization for all CCX–polymer combinations are summarized in Table 1.

CCX could be amorphized with VA64 and Soluplus probably due to the high drug solubility in these two polymers. Using XRPD, complete amorphization was achieved for the compact compositions CCX in VA64 or Soluplus after 180 s and 420 s, respectively. Compacts containing CCX in VA64 showed the overall fastest rate of amorphization (Table 1 and Figure 2a).

CCX displayed a low solubility at room temperature in HPMCAS, EPO, EL100, and PVA. However, the solubility of CCX in these polymers increased with increasing temperature, which in theory should allow in situ drug amorphization during laser exposure due to the elevated compact temperature during laser exposure. Indeed, CCX became fully amorphous in compacts containing HPMCAS and EPO after 420 s and 600 s, respectively (Table 1). However, no complete (or any) amorphization could be obtained for compacts containing CCX in EL100 and PVA even after 600 s of exposure to laser radiation (Table 1, Figure 2b and Figure S1). It should be noted that compacts containing HPMCAS, EPO, and Soluplus showed signs of recrystallization after 1.5–2 weeks, indicating the formation of a supersaturated ASD at room temperature (data not shown).

### 3.3. Temperature Measurements during Laser Exposure

**Figure 3:**
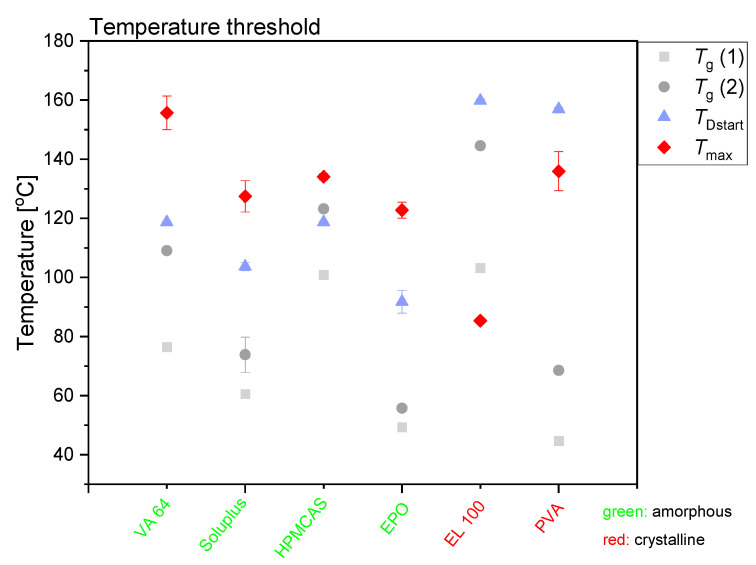
Schematic depiction for the definition of the temperature threshold for the different drug–polymer compact compositions. Compacts containing the green polymers became fully amorphous upon exposure to laser radiation and compacts containing the red polymers did not become fully amorphous. *T*_g_ 1 is the temperature of the *T*_g_ for the polymer with bulk water. *T*_g_ 2 is the temperature of the *T*_g_ for the water-free polymer. *T*_max_ is also shown in Figure 1. *T*_Dstart_ is determined from the drug–polymer solubility measurements. Mean ± SD (*n* = 2 for *T*_g_ 1, *T*_g_ 2, and *T*_Dstart_, *n* = 3 for *T*_max_).

It can be seen in Figure 1 and Figure 3 that different maximum compact temperatures were reached depending on the type of polymer utilized (Note: also after different exposure times). The individual temperature plots are shown in the Figure S2. The two polymers with the greatest difference in maximum compact temperature achieved during exposure to laser radiation were VA64 (*T*_max_ = 155.7 ± 5.7 °C) and EL100 (*T*_max_ = 85.4 ± 0.9 °C). The differences between the compact temperatures achieved upon exposure to laser radiation suggest that the compacts containing different polymers responded differently to the laser radiation.

From the maximum temperatures achieved during exposure to laser radiation and the solubility curves presented in Figure 1, it is theoretically possible to predict whether the maximum compact temperature obtained will allow a complete amorphization of the drug in the given polymer composition. The chosen drug load of 30 wt% CCX is clearly soluble in VA64 and Soluplus at the maximum compact temperatures, and complete amorphization can be expected. Due to the increased compact temperature upon exposure to laser radiation, the drug load of 30 wt% CCX can in theory also fully dissolve into HPMCAS and EPO at the maximum compact temperatures obtained according to the solubility curves (Figure 1). According to the CCX–polymer solubility, the temperature necessary to dissolve 30 wt% CCX in compacts containing HPMCAS is between 61 and 112 °C (Figure 1). Similarly, the temperature necessary to dissolve 30 wt% CCX in EPO is between 64 and 117 °C. The maximum compact temperature reached for compacts containing HPMCAS was 134.1 ± 1.1 °C (mean ± SD, *n* = 3). For compacts containing EPO, the maximum compact temperature was 122.8 ± 2.7 °C (mean ± SD, *n* = 3). Hence, the maximum compact temperatures for compacts containing HPMCAS and EPO were above the temperature necessary to dissolve 30 wt% CCX, and hence complete amorphization was obtained.

The chosen drug load of 30 wt% for EL100 compacts can, in theory, only be achieved at temperatures above 150–153 °C. Thus, at the maximum compact temperature achieved (*T*_max_ = 85.4 ± 0.9 °C), only a drug load of 1.5–2.4 wt% can be dissolved according to Figure 1. In accordance with this, little to no amorphization was observed upon exposure to laser radiation of CCX in EL100 (confirmed in Figure 2b). For PVA, the temperature necessary to dissolve 30 wt% CCX is between 117 and 157 °C, which was only reached (*T*_max_ = 135.9 ± 6.6 °C) at the longest exposure time (600 s). No complete amorphization was observed for CCX in PVA, possibly due to insufficient time at this temperature.

Not only did the different compact compositions reach different maximum compact temperatures, but the time of the initial heating rate and the time to reach the maximum compact temperatures were also significantly different (Figure S2). Compacts containing VA64, Soluplus, HPMCAS, EL100, and PVA showed a fast initial heating rate within the first 60 s of exposure, followed by a slower heating rate or even a temperature plateau. For compacts containing VA64 and PVA, the compact temperature increased steadily after the fast initial heating rate in the first 60 s. Compacts containing EPO showed a fast initial heating rate in the first 180 s followed by a temperature plateau. Comparing compacts containing EPO with compacts containing Soluplus, it was seen that the initial heating rate for compacts containing EPO was slower, i.e., the same compact temperature was reached after 180 s, compared to 60 s for compacts containing EPO and Soluplus, respectively (Figure S2).

With EL100, the maximum compact temperatures achieved were below 100 °C. It has previously been shown that an increase in PN load (from 0.1 wt% to 0.25 wt%) led to an increase in compact temperature [10]. In an attempt to reach a higher maximum compact temperature for compacts containing EL100 and PVA, the PN load was increased from 0.25 to 0.4 wt%. However, the maximum temperature reached upon exposure to laser radiation was not impacted with increasing PN load (data not shown). Thus, it seems that compacts with EL100 and PVA reached their maximum compact temperature at the laser intensity used, as all the light was already absorbed by 0.25 wt% PN.

Figure 3 summarizes the effect of the maximum compact temperature obtained during exposure to laser radiation on the amorphization of CCX. The temperature for the onset of the amorphization was determined by DSC analysis (*T*_Dstart_). As the amorphization follows a dissolution process, the dissolution of the drug is enhanced at the temperature of *T*_Dstart_, i.e., the viscosity has decreased enough to allow for drug dissolution in a measurable time frame. At temperatures below *T*_Dstart_, dissolution is possible if the drug is soluble at that temperature in the polymer; however, the dissolution rate will be so slow that it cannot be measured in the given time frame (kinetic hindrance due to high viscosity of the polymer). However, the dissolution process is a kinetic event, i.e., *T*_Dstart_ will be heating-rate dependent and increase with increasing heating rate and is therefore only an approximation. Nevertheless, the compact temperature must be at temperatures above *T*_Dstart_ to obtain a measurable drug dissolution in the given time frame. Furthermore, the temperature of *T*_Dstart_ is always above the *T*_g_ of the polymer. In fact, a significant decrease in viscosity of the polymer is often observed at approximately only 15–25 °C above the *T*_g_ of the polymer (determined by DSC) [23,25], allowing a drug dissolution process. In other words, the temperature of *T*_Dstart_ is at temperatures above approximately *T*_g_ + 20 °C. As most polymers used in this study contain sorbed water, the plasticized *T*_g_ (referred to as *T*_g_ 1) is particularly of interest (during in situ drug amorphization, small amounts of water will evaporate; hence, the practically relevant *T*_g_ will be somewhere between *T*_g_ 1 and *T*_g_ 2 (water-free *T*_g_)) (see Table 2).

From Figure 3 it can be seen that for all compacts that became fully amorphous upon exposure to laser radiation, *T*_max_ was above *T*_Dstart_, i.e., the reached maximum compact temperature allowed for fast dissolution of the drug into the mobile polymer. Conversely, for the compacts that did not become fully amorphous upon exposure to laser radiation, *T*_Dstart_ was above *T*_max_. Therefore, no amorphization was possible in the given time frame. Even though CCX has a solubility of 34.0 wt% (t_2.5_ = 15.3 wt%, t_97.5_ = 53.8 wt%) at 140 °C in PVA (maximum compact temperature reached, see Figure 3), no complete amorphization was seen, as the *T*_max_ was below *T*_DStart_ (Figure S2 and Figure 3). It is suggested that the nonconsistent decrease in peak intensity in the XRP-diffractograms (see Figure S1) originated from CCX degradation rather than amorphization (see also Section 3.4.).

### 3.4. HPLC Data

Firstly, the amount of CCX in the different compacts was determined prior to exposure to laser radiation. The amount incorporated inside the compacts was detected, i.e., the polymers did not interfere with the detection and quantification of CCX. It was possible to quantify the 30 wt% CCX in all compact compositions before exposure to laser radiation. Secondly, the CCX amount incorporated inside the compacts was also detected after exposure to laser radiation for compacts containing VA64, Soluplus, HPMCAS, EPO, and EL100 at the maximum exposure time to laser radiation (data not shown), i.e., 30 wt% CCX was detected inside the sample injected into the HPLC column. In contrast, for the compact containing PVA exposed for 600 s to laser radiation, CCX could only partly be detected (one sample showed only 11 wt% CCX), whilst others contained 28–30 wt% CCX (as incorporated). Visual inspection of the HPLC elution profiles showed a slight increase of the peak height and AUC of the degradation products of CCX in some cases, though not all. It remains unclear if degradation was the cause of the loss of CCX inside compacts containing PVA after exposure to laser radiation. PVA was, however, not a suitable polymer for laser-induced in situ amorphization of CCX due to the maximum temperature reached being below *T*_DStart_.

## 4. Conclusions

This study showed that successful in situ drug amorphization upon exposure to laser radiation was possible with a range of different pharmaceutically relevant polymers. Using low amounts of PNs (0.25 wt%) in compacts containing CCX and polymer, complete amorphization was possible for a drug load of 30 wt% in VA64, Soluplus, HPMCAS, and EPO. Complete amorphization was not achieved for CCX in EL100 and PVA. Different rates of amorphization, due to different heating rates and maximum compact temperatures, were obtained during exposure to laser radiation for the different polymers. It was found that for a successful laser-induced in situ drug amorphization, it is important to obtain temperatures above that of the onset of the dissolution (*T*_Dstart_) of the respective drug–polymer composition. Hence, laser-induced in situ drug amorphization is suitable for polymers in which the drug is soluble and for which compact temperatures above *T*_Dstart_ can be reached.

## Figures and Tables

**Table 1:** Exposure times (s) of the different compact compositions.

Compact Composition	60 s	120 s	180 s	240 s	300 s	360 s	420 s	480 s	600 s
VA64	c	c	a						
Soluplus	c	c	c	c	c	c	a		
HPMCAS	c	c	c	c	c	c	a		
EPO	c	c	c	c	c	c	c	c	a
EL100									c
PVA			c		c		c		c

c: Indicates residual crystallinity detected by XRPD. a: Indicates amorphization detected by XRPD. Note: Compacts containing HPMCAS, EPO, and Soluplus were not stable and showed signs of recrystallization after 1.5–2 weeks under nonspecific storage conditions; this indicates the formation of a supersaturated ASD at room temperature.

**Table 2:** Glass transition temperatures (*T*_g_) (°C) of the polymers.

Polymer	*T*_g_1	*T*_g_2
VA64	76.5 ± 0.4	109.1 ± 0.1
Soluplus	60.6 ± 0.0	73.8 ± 6.0
HPMCAS	100.9 ± 1.0	123.2 ± 0.0
EPO	49.4 ± 0.6	55.8 ± 0.8
EL100	103.2 ± 0.0	144.5 ± 0.9
PVA	44.7 ± 0.1	68.5 ± 0.0

*T*_g_1: Containing water (bulk polymer); *T*_g_2: water-free. Mean ± SD (*n* = 2).

## Data Availability

Data can be requested from the authors.

## Supplementary Materials

The following are available online at https://www.mdpi.com/article/10.3390/pharmaceutics13060917/s1, Table S1: Drug–polymer solubilities at room temperature, Figure S1: XRP-diffractograms, Figure S2: Temperature measurements.
