# Supplementary material for: The Influence of Drug–Polymer Solubility on Laser-Induced In Situ Drug Amorphization Using Photothermal Plasmonic Nanoparticles
Source: Pharmaceutics. 2021 Jun 21;13(6):917. doi: 10.3390/pharmaceutics13060917 (PMC8234654; doi:10.3390/pharmaceutics13060917)
Supplement: Supplementary file 1 [file pharmaceutics-13-00917-s001.zip › pharmaceutics-1236049-SI.pdf]

pharmaceutics

Supplementary Materials: The Influence of Drug–Polymer Solubility on Laser-Induced In Situ Drug Amorphization Using Photothermal Plasmonic Nanoparticles

Nele-Johanna Hempel, Padryk Merkl, Matthias Manne Knopp, Ragna Berthelsen, Alexandra Teleki, Georgios A. Sotiriou and Korbinian Löbmann

S.1. Drug-Polymer Solubility

The respective graphs for each drug-polymer combination are shown in Figure 1.

Table S1. Solubility of CCX at 20 °C in the six different polymers given with the confidence interval.

Polymer	Solubility [wt%]	$t_{2.5}$ [wt%]	$t_{97.5}$ [wt%]
VA64	31.8	27.6	35.0
Soluplus	22.5	7.3	34.6
HPMCAS	5.3	1.1	13.6
EPO	3.6	0.6	11.9
EL100	0	0	0
PVA	0	0	0

S.2. XRPD Analysis

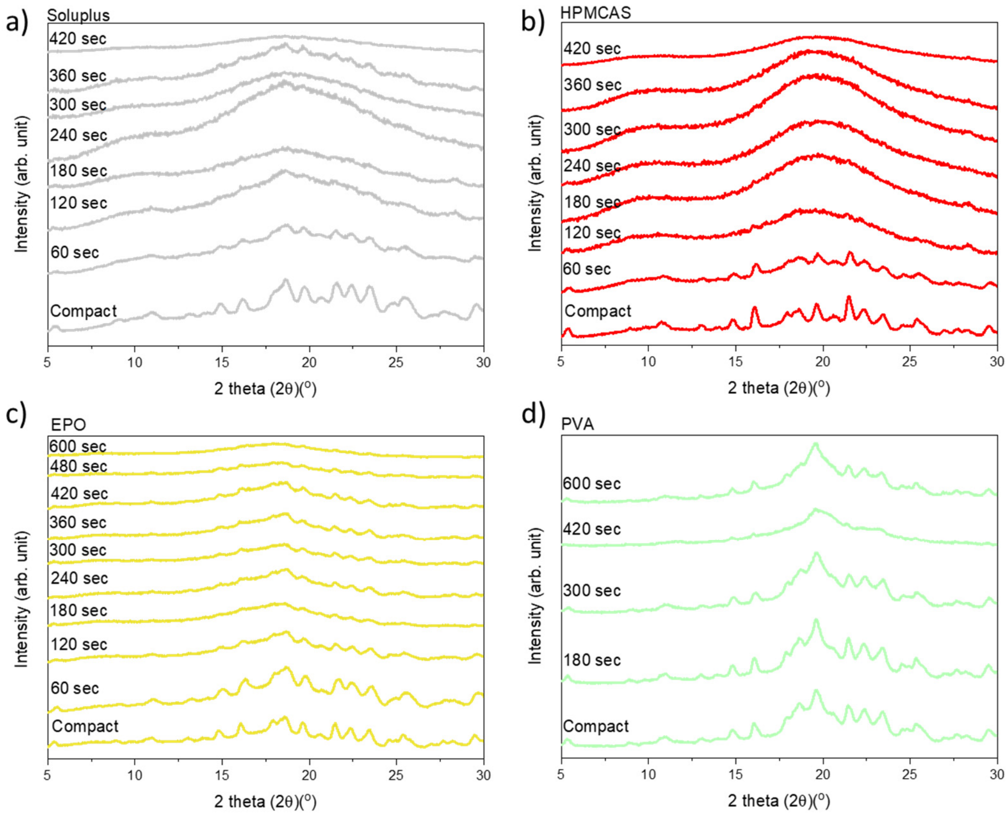

Figure S1. XRPD analysis after different exposure times to laser radiation [sec] and before exposure to laser radiation (compact) for the different compact compositions. **a**) 30 wt% CCX in Soluplus; **b**) 30 wt% CCX in HPMCAS; **c**) 30 wt% CCX in EPO; **d**) 30 wt% CCX in PVA. Note: Due to the scaling of the diffractograms, it is sometimes difficult to see small peaks in the figures. The remaining diffractograms are part of the main manuscript.

S.3. Temperature Measurements

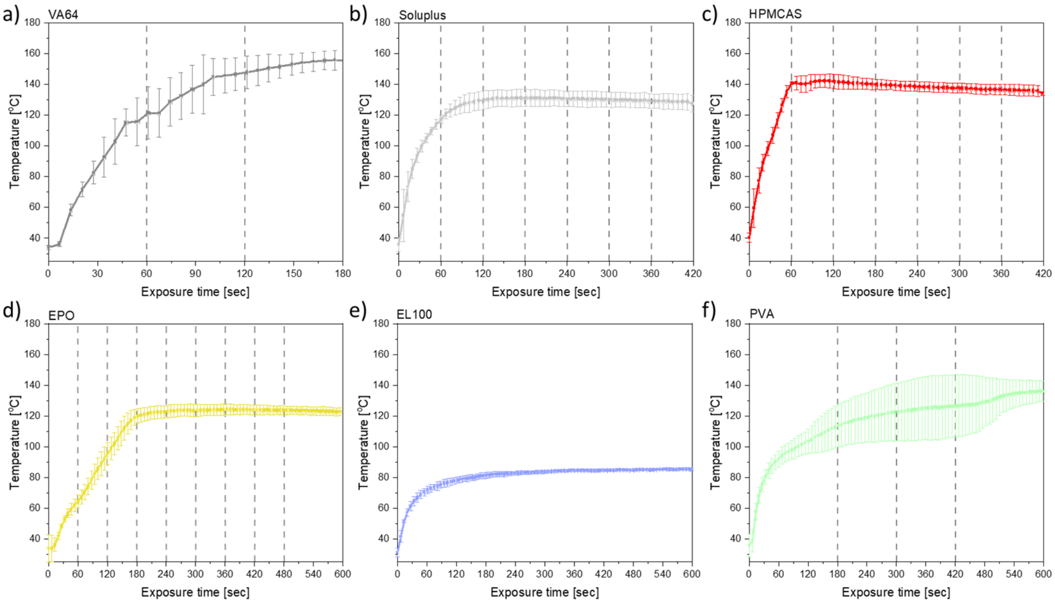

Figure S2. Temperature measured [°C] during exposure to laser radiation [sec] for the different compact compositions. **a**) 30 wt% CCX in VA64; **b**) 30 wt% CCX in Soluplus; **c**) 30 wt% CCX in HPMCAS; **d**) 30 wt% CCX in EPO; **e**) 30 wt% CCX in EL100; **f**) 30 wt% CCX in PVA. The dashed line indicate the different exposure times for XRPD analysis. Mean ± SD (*n*=3).
